# Interleukin-10 promoter polymorphism predicts initial response of chronic hepatitis B to interferon alfa

**DOI:** 10.1186/1743-422X-8-28

**Published:** 2011-01-20

**Authors:** Shaoyang Wang, Dedong Huang, Shunlai Sun, Weimin Ma, Qin Zhen

**Affiliations:** 1 Department of infectious diseases, the Fuzhou General Hospital, Fu Zhou, Fujian Province 350003, China

## Abstract

In order to examine whether variation in interleukin-10 promoter polymorphism would predict the likelihood of sustain response of chronic hepatitis B to treatment with interferon alfa (IFN-α), the inheritance of 3 biallelic polymorphisms in the IL-10 gene promoter in patients with 52 chronic hepatitis B were determined by polymerase chain reaction (PCR)-bared techniques, restriction enzyme digestion or direct sequencing. The relationship to the outcome of antiviral therapy for chronic HBV infection was studied in 24 patients who had a virologically sustained response(SR) and in 28 non-responder(NR) to interferon alfa-2b and several IL-10 variants were more frequent among SR compared with NR. Carriage of the -592A allele, -592A/A genotype and -1082/-1819/-592 ATA haplotype was associated with SR. Our findings indicate that heterogeneity in the promoter region of the IL-10 gene has a role in determining the initial response of chronic hepatitis B to IFN-α therapy.

## Introduction

Hepatitis B is a worldwide disease and remains a significant etiology of chronic hepatitis, cirrhosis and hepatocellular carcinoma, especially in several areas of Asia and Africa[1]. It is estimated to affect over 350 million people worldwide, with a mortality of over 1.2 million deaths per year because of acute or chronic hepatitis B infection[2,3]. For active hepatitis B patients with detectable hepatitis B virus e antigen (HBeAg) or hepatitis B virus (HBV) DNA and elevated alanine aminotransferase (ALT) serum levels, treatment is often recommended. Six-month course of interferon alfa (IFN-α) therapy has been shown to induce a long-term sustained remission in 25% to 40% of chronic hepatitis B patients[1,4,5]. However, the question remains unresolved as to why only a certain percentage of patients respond to therapy. Hence, predictive factors determining therapeutic responses are focused by many investigations.

Multivariate analyses have shown that the most important predictors of good response to IFN-αtreatment include high ALT levels, low serum HBV DNA, female gender, and histological activity on liver biopsy in chronic HBV patients[6-8]. However, despite these studies of viral factors and clinical markers affecting treatment response, the role of the host genetic background was less well studied[9].

The role of cytokines and the cellular immune response in the pathogenesis and eradication of chronic HBV has been investigated. Several proinflammatory cytokines such as Th1 cytokines (including IL-2 and IFN-γ) and TNF-α are believed to participate in elimination of HBV [8,10,11]. In contrast, IL-10 and IL-4, Th2 cytokine, act as potent inhibitors of Th1 effectors mechanisms[8,12-14]. There are some evidences that the capacity for cytokine production in individuals has a major genetic component [15]. This has been ascribed to polymorphisms within the regulatory regions or signal sequences of cytokine. Several polymorphic sites within the IL-10 gene promoter region have been described, including three bi-allelic polymorphisms at positions--1082, --819, and --592 from the transcription start site. The IL-10--819 T and C alleles were completely in linkage disequilibrium with the IL-10--592A and C alleles, respectively. The--592A allele was exclusively associated with the--1082A allele. These result in three different haplotypes: GCC, ACC, and ATA[16]. It was reported that allelic variation in these polymorphisms may be associated with the disease progression of chronic HBV infection[17]. Heterogeneity in the promoter region of the IL-10 gene has been reported to have a role in determining the initial and sustained response of chronic hepatitis C to IFN-αtherapy[18]. However, there are differences in the immunopathogenesis of HBV and HCV infection[19], it is necessary to investigate whether IL-10 gene promoter polymorphisms could serve as a candidate prediction of response to IFN-αtherapy in chronic HBV infection. To prove this hypothesis, we examined the inheritance of the 3 biallelic polymorphisms in patients with chronic HBV and the association of these polymorphisms with response to IFN-α. For HBV patients, it is very important to predict the response to antiviral therapy, especially for IFN-α therapy, given the many displeasing side effects associated with this medical regimen and the high cost of therapy.

## Patients and methods

### Patients

We retrospectively enrolled 52 Chinese Han patients with chronic hepatitis B from our outpatients clinics at Fuzhou general hospital, between February 2007 and December 2008. There were 28 non-responders (NR) to IFN treatment with a mean age of 32 years and 24 sustained responders (SR) with a mean age of 35 years. Males outnumbered females (M:F/40:16). All patients' blood samples were hepatitis B virus surface antigen (HBsAg) positive and HBeAg positive and with an elevated ALT of at least 2-fold higher than the upper limits of normal for 6 months. ALTs of SR group and NR group were 180.3 ± 54.5 U/L and 197.2 ± 75.5 U/L respectively. ALT was no significant difference between the SR and NR (*P *= 0.354). Log_10_HBV DNAs of the two groups were 6.06 ± 8.3 copies/ml and 6.3 ± 8.2 copies/ml respectively. It was no significant difference between the two groups (*P *= 0.284). Patients were excluded from receiving IFN-αtherapy if they had any of the following criteria: neutrophil count < 1,500 cells/mm^3^, Hgb < 110 g/L in women or 120 g/L in men, or platelet count <90 cells/L, history of poorly controlled thyroid disease, and serum creatinine level >1.5 times the upper limit of normal at screening. Eligible patients received IFN-α(2b) at a dosage of 5 million units (MU) 3 times per week for 6 months and were subsequently followed for treatment response via clinical, biochemical, and serologic markers for more than 1 year. The definition of sustained SR to IFN-αtreatment for chronic hepatitis B disease included patients with HBeAg(+) to HBeAg(-) conversion and HBVDNA level <1000 copies/ml after treatment for at least 1 year after follow-up. NR were those with persistent or relapsed HBeAg(+) and HBVDNA level >1000 copies/ml during the follow-up period. Patients coinfected with hepatitis C or D were excluded from the study. In addition, 48 healthy volunteers (31 men and 17 women, a mean age of 33 years), were enrolled as a control group. Informed consent was obtained from each patient, and the study protocol was approved by the Fuzhou general Hospital Ethics Committee.

### DNA extraction

Genomic DNA was extracted from a 5 ml sample of whole blood collected into EDTA. Extraction was performed using a commercial kit (Omega, USA) according to the manufacturer's instructions.

### IL-10 Genotyping

The 3 biallelic IL-10 promoter polymorphisms were detected by PCR using primers that amplified a short fragment of DNA containing the polymorphism (Table 1).

**Table 1:** Identification of the 3 biallelic IL-10 promoter polymorphisms by PCR amplification and restriction enzyme digest

IL-10 promoter polymorphism (from transcription initiation site)	-592 (A/C)	-819 (T/C)	-1082 (G/A)
PCR primers	5' cct agg tca cag tga cgt gg 3' 5' ggt gag cac tac ctg act agc 3'	5' tca ttc tat gtg ctg gag atg g 3' 5' tgg ggg aag tgg gta aga gt 3'	5' ctc gct gca acc caa ctg gc 3' 5' ctc gct gca acc caa ctg gc 3'
PCR product size (bp)	419	419	139
Restriction enzyme	*Rsa *1	*Mae *III	*Mnl*
Digest interpretation	Cuts the rarer A allele to generate 176- and 236-bp fragments	Cuts the more common C allele to generate242- and 141-bp fragments	direct sequencing

**Figure 1:**
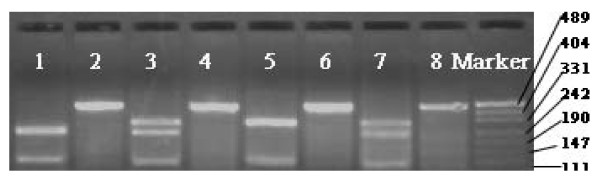
**Representative agarose gel electrophoresis illustrating PCR products for the IL-10 promoter polymorphisms(-592 polymorphism): lane 2, 4, 6 and 8, 456 bp marker; lane 1, homozygous AA subject; lane 3 and 7, heterozygous subject; lane 5, homozygous CC subject, C allele does not cut with Rsa 1; A allele cuts with Rsa 1 to generate 240- and 115-bp fragments**.

**Figure 2:**
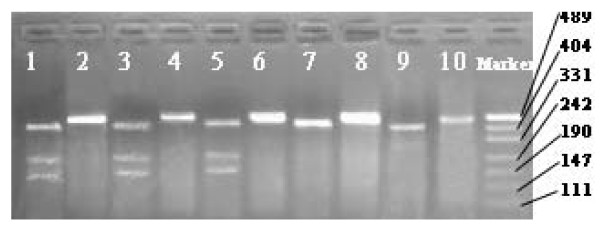
**Representative agarose gel electrophoresis illustrating PCR products for the IL-10 promoter polymorphisms(-819 polymorphism): lane 2,4,6, 8 and10, 456 bp marker; lane 1,3 and 5, heterozygous subject, lane 7 and 9, homozygous TT subject, T allele does not cut. C allele cut with Mae III generating 212- and 179-bp fragments**.

**Figure 3:**
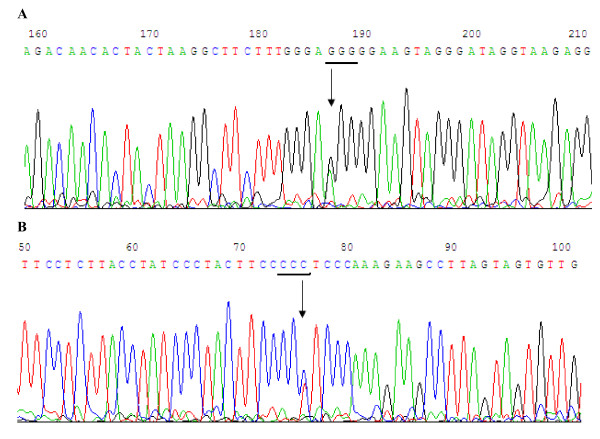
**The sequence of -1082 ballelic in IL-10 promoter**. **A**. The -1082 ballelic in IL-10 promoter polymorphism was sequenced by upstream primer of 1082 ballelic. As shown on the figure 187 bp is the site of -1082 bp in IL-10 promoter. There were A and G.. **B**. The -1082 ballelic in IL-10 promoter polymorphism was sequenced by downstream primer of 1082 ballelic. As shown on the figure 76 bp is the site of -1082 bp in IL-10 promoter. There were T and C.

Amplification of the -592 fragment was performed in a volume of 25 μL containing 250 ng of template DNA, 10 mmol/L Tris-HCL (pH 8.3), 50 mmol/L KCl, 1.5 mmol/L MgCl_2 _, 0.8 mmol/L deoxyribonucleotides, 0.5 μmol/L of each primer, and 0.6 U AmpliTaq DNA Polymerase (Takara, DaLian, China). The parameters for amplification of the -819 fragment were the same except that a final concentration of 2 mmol/L MgCl_2 _was used. Amplification of the -1082 polymorphism was performed using the TakaraTaq kit (Takara, DaLian, China), and Q-Solution was included in the PCR reaction mix. The parameters for thermocycling were as follows: denaturation at 94°C for 2 minutes, followed by 35 cycles of denaturation at 94°C for 30 seconds; annealing at 56°C for 30 seconds; and extension at 72°C for 4 minutes 30 seconds. This was followed by final extension at 72°C for 5 minutes. Identification of the 2 alleles at each polymorphic site was performed by incubating PCR product with a restriction enzyme chosen to cut 1 of the 2 alleles (Table1), followed by electrophoresis on agarose gels (3%) (Figure 1 and Figure 2), All samples were amplified and digested in parallel with 2 samples of known genotype and water. IL-10 -1082 polymorphism performed using direct sequencing because fragment of alleles was not identified clearly after restriction enzyme digestion(Figure 3).

### Statistical Analysis

Genotype frequencies of each single nucleotide polymorphism(SNP) between SR and NR were compared by Fisher Probability. Multiple logistic regression was performed to evaluate whether there was a difference in response effect for each SNP after adjustment for age, sex, and HBV DNA level. All statistical tests were 2-tailed. *P *values less than 0.05 were considered statistically significant. The analyses were performed by the SPSS statistical package version 16.

## Results

### Polymorphisms of the IL-10 promoter in patients with HBV and healthy volunteers

Three biallelic polymorphisms and genotype/haplotype frequencies in the IL-10 gene promoter were analyzed (Table 2). The majority of HBV carriers as well as healthy volunteers had A allele at position -1082 and T allele at position -819 in the IL-10 gene promoter. In addition, there was no significant difference in the frequencies of alleles or genotype/haplotype in the IL-10 gene promoter between HBV carriers and healthy volunteers.

**Table 2:** Frequencies of IL-10 Promoter Alleles and Genotype/Haplotypes in Patients With HBV and healthy Control Population

	patients (%)	control (%)	OR	*P*
Allele	n = 52 × 2	n = 48 × 2		
- 1082G	4(3.9)	3(3.1)	1.24	0.99
- 1082A	100(98)	93(96.8)	0.81	0.99
- 819C	18(17.3)	18(18.7)	0.91	0.85
- 819T	86(92.7)	78(81.3)	1.97	0.85
	38(36.5)	22(22.9)	1.88	0.05
- 592A	66(63.4)	74(77.1)	0.52	0.05
Genotype	n = 52	n = 48		
- 592 A/A	23 (44.2)	30 (62.5)	0.48	0.08
A/C	20(38.4)	14(29.2)	1.52	0.40
C/C	9(17.3)	4(8.3)	2.3	0.24
- 819T/T	36(69.2)	33(68.8)	1.02	0.99
T/C	12(23.1)	12(23.1)	0.9	0.99
C/C	4(7.7)	4(7.7)	1.25	0.99
- 1082 A/A	48(92.3)	45(93.8)	0.8	0.99
A/G	4(7.7)	3(6.3)	1.25	0.99
G/G	0(0)	0(0)		
Heplotype(-1082/-819/-592)				
	n = 52	n = 48		
GCC	4 (7.6)	3(6.2)	1.25	0.99
ACC	13 (25)	14(29.1)	0.81	0.66
ATA	41 (78.8)	43(89.5)	0.43	0.18
GTA	2 (3.8)	2(4.1)	0.92	0.99

NOTE: n is the number of patients or controllers. *P *values were caculated by Fisher Probability. *P *values less than 0.05 were considered statistically significant. SR: sustained response; NR: non-responders.

### Association of IL-10 gene promoter polymorphisms with initial response to IFN-α therapy in patients with HBV

Fifty-two patients received treatment with IFN-α (5 million units, 3 times weekly) for 6 month. Twenty-eight patients were classified as "nonresponders" as a result of persistent or relapsed HBeAg(+) and HBVDNA level >1000 copies/ml during the follow-up. HBeAg(+) to HBeAg(-) conversion and HBVDNA level <1000 copies/ml after treatment for at least 1 year after follow-up was seen in Twenty-four patients ("responders").

Differences between SR and NR in several IL-10 allele, genotype, and haplotype distributions were observed. The -592A and -819T alleles, along with the exclusively linked -592A/A and -819T/T genotypes (Table 3), were more frequent in SRs than in NRs. These two sites are dimorphic, and reciprocal effects (nonresponse) were also seen with the -592C and -819C alleles. Homozygosity for genotypes -592A/A and -819T/T was more strongly associated with sustained response than heterozygosity. Similarly, inheritance of the haplotype ATA was associated with "responder" status to IFN-**α**therapy.

**Table 3:** IL-10 Gene Promoter Polymorphisms With Initial Response to IFN-α therapy in patients with HBV

	SR(%)	NR(%)	OR	*p*
Allele	n = 24 × 2 = 48	n = 28 × 2 = 56		
-819C	4(8.3)	16(28.6)	0.23	0.01
-819T	44(91.6)	40(71.4)	4.4	0.01
-592C	8(16.6)	32(57.1)	0.15	<0.01
-592A	40(83.3)	24(42.9)	6.67	<0.01
-1082A	47(97.9)	53(94.6)	2.66	0.62
-1082G	1(2.1)	3(5.4)	0.38	0.62
Genotype	n = 24	n = 28		
-592A/A	19(79.1)	4(14.3)	22.8	<0.01
-592A/C	2(8.3)	18(64.3)	0.05	<0.01
-592C/C	3(12.5)	6(21.4)	0.52	0.48
-819T/T	21(87.5)	15(53.6)	6.07	0.02
-819T/C	2(8.3)	10(35.7)	0.16	0.02
-819C/C	1(4.2)	3(10.7)	0.36	0.62
-1082A/A	23(95.8)	25(89.3)	2.78	0.62
-1082A/G	1(4.2)	3(10.7)	0.36	0.62
-1082G/G	0(0)	0(0)	-	-
Heplotype	n = 24	n = 28		
ATA	19(79.1)	13(46.4)	4.38	0.02
GCC	0(0)	3(10.7)	-	-
ACC	5(20.8)	12(42.8)	0.35	0.14

NOTE: n is the number of patients or controllers. P values were caculated by Fisher Probability. P values less than 0.05 were considered statistically significant. SR: sustained response; NR: non-responders.

## Discussion

The host genetic factors involving genetic polymorphisms are believed to be responsible for clinical outcomes of infectious disease[9,17,19], because differences in the susceptibility to infection or severity of disease cannot solely be attributed to the virulence of an organism. For chronic viral hepatitis, genetic associations are likely to provide some clues to viral persistence and disease progression, and might lead to a new therapeutic approach. Recent studies have shown that several immunoregulatory cytokines such as IFN-γand TNF-α inhibit HBV replication through the noncytolytic process[20]. In contrast, IL-10 counteracts their effector mechanisms[8,10,11,17]. Because the capacity for cytokine production in individuals largely depends on genetic polymorphisms[21], heterogeneity of the candidate gene in patients with HBV emerges as a probable biomarker for determining the disease phenotypes.

In HCV infection, the influence of IL-10 genotypes either on different clinical features of liver disease or in the response to antiviral therapy has been evaluated in several studies: data are highly controversial with some studies showing a positive association and others denying such a link[18,22,23]. Taken together, the some investigation has shown that responsiveness to IFN-αtreatment in patients with chronic hepatitis C is closely linked to ATA haplotype of the IL-10 gene promoter. For example Edwards-Smith et al. showed an association of the IL-10 (-592) CC genotype with NR and ATA haplotype with SR. Although IL-10 has both anti-inflammatory and antifibrotic properties, high levels of IL-10 production may increase viral replication in chronic HBV infection and result in influence of the immune response, moreover, there are differences in the immunopathogenesis of HBV and HCV infection[19]. So the association between IL-10 promoter polymorphism and the response to IFN-αtherapy in HBV infection may be evaluated.

In our study, when comparing HBV-infected patients with healthy volunteers, no different distribution of the three cytokine genotypes was observed. It demonstrates the patients and healthy controls share an identical genetic background and the cytokine polymorphisms do not influence susceptibility to the HBV infection.

Our results also indicate that inheritance of particular IL-10 promoter genotypes/haplotypes has a significant role in determining the initial response of HBV infection to treatment with IFN-α. Inheritance of the -592A and -819T alleles, along with the exclusively linked -592A/A and -819T/T genotypes or the ATA haplotype (lower IL-10 producer) were significantly associated with "responder" status. Two sites are dimorphic, and reciprocal effects (nonresponse) were also seen with the -592C and -819C alleles. Homozygosity for genotypes -592A/A and -819T/T was more strongly associated with sustained response than heterozygosity.

As a potent immune modulator, IL-10 may exert a profound impact on the overall therapeutic outcome in patients with HBV. High serum levels of IL-10 have been correlated with poor response to interferon therapy, whereas IL-10 production has been found to be lower in responders than in nonresponders[14,19]. Both CD4+ T-helper cell and CD8+ cytotoxic T-lymphocyte (CTL) responses are important in HBV infection. If IL-10 operates through T-lymphocyte pathways, the exact mechanism of action may be complicated. IL-10 may down-regulate MHC class I and class II expression, impeding both CTL and antibody responses, but may enhance natural killer cell activity[24,25]. Strong anti-HBV-specific T-helper response may contribute to self-limiting HBV infection and sustained response to interferon therapy, and similar effects can be attributed to HBV-specific CTL response [26,27].

The mechanism of IL-10 Promoter Polymorphism and the HBV infection sustained response to interferon therapy need to further study. If serum IL-10 were detected in the SR and NR, this study would give more evidence. It may be interesting to investigate the promoter of IL-10 polymorphisms and HBV patients initial response of chronic hepatitis B to IFN-α therapy in Caucasians patients.

In summary, Our findings indicate that heterogeneity in the promoter region of the IL-10 gene has a role in determining the initial response of HBV infection to IFN-α therapy. Patients who are genetically predisposed to high IL-10 production have a poor response to IFN-α and may benefit from additional treatment strategies designed to enhance a Th1 response in the meantime. Identifying other predictors, especially host genetic factors, for treatment outcome in these patients may help in making appropriate treatment decisions.

## Competing interests

The authors declare that they have no competing interests.

## Authors' contributions

SW conceived of the study, and participated in its design and coordination. DH drafted the manuscript and performed the statistical analysis. SS carried out the molecular genetic studies and drafted the manuscript. WM participated in the collecting of the clinical data. QZ participated in the design of the study. All authors read and approved the final manuscript.
